# A stakeholder-driven approach to designing a peer recovery coach role for implementation in community-oriented primary care teams in South Africa

**DOI:** 10.1186/s13722-025-00544-3

**Published:** 2025-02-13

**Authors:** Bronwyn Myers, Kristen S. Regenauer, Kim Johnson, Imani Brown, Alexandra L. Rose, Nonceba Ciya, Sibabalwe Ndamase, Yuche Jacobs, Morgan S. Anvari, Abigail Hines, Dwayne Dean, Rithika Baskar, Jessica F. Magidson

**Affiliations:** 1 Curtin enAble Institute, Faculty of Health Sciences, Curtin University, Kent Street, Bentley, WA Australia; 2 Mental Health, Alcohol, Substance Use, and Tobacco Research Unit, South African Medical Research Council, Parow, Cape Town, South Africa; 3 West Australian Country Health Service and Curtin University Research and Innovation Alliance, Perth, WA Australia; 4 Department of Psychology, University of Maryland, College Park, College Park, MD USA; 5 Center for Substance Use, Addiction & Health Research (CESAR), University of Maryland, College Park, College Park, MD USA

**Keywords:** Stigma, Substance use, Implementation science, Task-sharing, Low-and-middle income country, Global mental health, Lived experience, Co-design

## Abstract

**Introduction:**

In South Africa, community-oriented primary care teams work to re-engage out-of-care people with HIV (PWH) in treatment, many of whom have substance use (SU) concerns. SU stigma is high among these teams, limiting care engagement efforts. Integrating peer recovery coaches into community-oriented primary care teams could shift SU stigma and improve patients’ engagement in care. The peer role does not exist in SA and represents a workforce innovation. To enhance acceptability, feasibility, and appropriateness for the local context, we engaged multiple stakeholder groups to co-design a peer role for community-oriented primary care team integration.

**Methods:**

We used a five-step human-centered design process: (i) semi-structured interviews with healthcare worker (*n* = 25) and patient (*n* = 15) stakeholders to identify priorities for the role; (ii) development of an initial role overview; (iii) six ideation workshops with healthcare worker (*n* = 12) and patient (*n* = 12) stakeholders to adapt this overview; (iv) refinement of the role prototype via four co-design workshops with healthcare worker (*n* = 7) and patient (*n* = 9) stakeholders; and (v) consultation with HIV and SU service leaders to assess the acceptability and feasibility of integrating this prototype into community-oriented primary care teams.

**Results:**

Although all stakeholders viewed the peer role as acceptable, patients and healthcare worker identified different priorities. Patients prioritized the care experience through sharing of lived experience and confidential SU support. Healthcare worker prioritized clarification of the peer role, working conditions, and processes to limit any impact on the community-oriented primary care team. A personal history of SU, minimum 1 year in SU recovery, and strong community knowledge were considered role prerequisites by all stakeholders. Through the iterative process, stakeholders clarified their preferences for peer session structure, location, and content and expanded proposed components of peer training to include therapeutic and professional work practice competencies. Service leaders endorsed the prototype after the addition of peer integration training for community-oriented primary care teams and peer mentoring to address community and team dynamics.

**Conclusion:**

Stakeholder engagement in an iterative design process has been integral to co-designing a peer role that multiple stakeholder groups consider acceptable and that community-oriented primary care teams are willing to implement. This offers a methodological framework for other teams designing SU workforce innovations.

## Background

Although South Africa has significantly expanded access to antiretroviral therapy (ART) for people with HIV (PWH), less than two-thirds of PWH in South Africa attain viral suppression [1]. Intermittent or discontinued HIV treatment contributes to sub-optimal viral suppression rates [2], with only 38% of PWH continually engaging in ART during the first 12 months after initiation [3].

In response, the South African Department of Health has introduced ward- or community-oriented primary care teams to bridge gaps between out-of-care patients and clinic-based HIV services [4, 5]. These teams are linked to a primary care clinic and comprise an enrolled nurse outreach team leader and community health workers (CHW) [6]. Community-oriented primary care teams provide up to 400 households in their clinic’s geographical catchment area with HIV treatment supports, including support for out-of-care PWH to re-engage with clinic-based services and referral to health and social services for co-occurring conditions that affect HIV treatment engagement [6, 7]. One co-occurring condition that CHWs are likely to encounter when working with out-of-care PWH is substance use (SU). SU is highly prevalent in South Africa [8], particularly in the Western Cape province where population-level rates of SU disorders are significantly higher than the rest of the country [9]. Alcohol, cannabis and methamphetamine are the main substances for which people seek treatment in the Western Cape [10, 11]. Although the prevalence of SU among PWH is unknown, approximately a third of PWH attending clinics in the Western Cape are estimated to have SU difficulties [12]. As SU is associated with sub-optimal ART adherence and treatment disengagement [13, 14], the prevalence of SU difficulties may be even higher among out-of-care PWH being served by community-oriented primary care teams.

Yet CHWs are not routinely trained to screen for SU, limiting their ability to assist out-of-care PWH struggling with this care engagement barrier and to identify patients who may benefit from referral to SU treatment [15–17]. This is a missed opportunity to support PWH who use substances, particularly given recent investments in scaling access to SU treatment in many HIV-affected communities in the Western Cape [18], although there has been relatively low uptake of these services by PWH [19].

More specifically, the Western Cape has a relatively well-resourced, diversified SU treatment system compared to the rest of South Africa [11, 18]. There are approximately 40 treatment sites, mainly dispersed across Cape Town’s eight health subdistricts. Most of these SU treatment facilities offer outpatient or intensive outpatient services; residential services are less common. As these facilities are primarily operated by non-governmental organizations (NGOs) funded by the National Department of Social Development or by provincial or local government, they offer free or low-cost treatment. A private-for-profit SU treatment sector also exists but treatment costs make these services largely inaccessible to HIV-affected communities [18, 20]. Non-profit and government services treat all types of substance use disorders and are required to provide evidence-based behavioral treatments like motivational enhancement therapy, cognitive behavioral treatment, or the Matrix model. Access to pharmacotherapy is limited to individuals who can afford these medications [11, 18].

While there have been recent initiatives to support referral to these services by training CHWs and other healthcare workers to screen for SU, and provide brief interventions and referral to treatment [21], high levels of SU stigma among CHWs [22, 23] may limit the potential benefits of this training for both HIV care engagement and SU treatment initiation.

SU stigma among CHWs and other healthcare workers affects access to effective HIV care [22–24] and SU treatment initiation [25, 26]. Evidence suggests that healthcare workers with high levels of SU stigma are less likely to provide evidence-based interventions or person-centered care [27–30]. In addition, anticipated and enacted SU stigma affects PWH’s readiness for HIV and SU care [31–33]. Therefore, SU stigma needs to be addressed for CHWs to effectively support people with SU to re-engage in HIV treatment and to overcome barriers to SU treatment.

Converging evidence suggests that stigma reduction interventions involving sustained social contact with people in SU recovery have the largest and most durable effects on healthcare worker stigma [28, 34]. Consequently, peer recovery coaches (hereafter referred to as peers) —trained individuals with lived experience of SU—are increasingly being integrated into primary healthcare teams in the US as a strategy for expanding their capacity to link patients with SU to care [35, 36]. In these teams, peers work directly with patients to provide personalized support for navigating barriers to accessing and engaging in SU treatment and other needed health care as well as personalised interventions to support SU behavior change and facilitate SU recovery navigation. US-based peer intervention studies have demonstrated the peer role’s feasibility and acceptability, reporting significantly higher rates of SU treatment initiation and engagement among patients who received peer-delivered supports compared to those in standard care [36–40].

Integrating peers into community-oriented primary care teams may help shift SU stigma among CHWs and may offer PWH direct support to overcome SU-related barriers to HIV care engagement while helping them navigate obstacles to accessing SU treatment and for SU behavior change and recovery [22]. In fact, the idea of training peers to provide SU-related supports for PWH emerged organically from our team’s prior work with PWH and lived experience of SU in this setting [23]. Despite this, the peer role represents a workforce innovation for SA, and there are likely to be patient-, provider-, and system-level barriers to the uptake, implementation, and sustainment of this new role. To optimize acceptability, feasibility and contextual appropriateness, we partnered with key stakeholders and used a human-centered design process [41, 42] to develop a peer role that builds off successful US models. This was informed by Designing for Dissemination and Sustainability, an implementation science concept that recommends stakeholder engagement in the design process as a strategy for ensuring that innovations are developed that are aligned with stakeholder preferences and priorities and responsive to potential implementation barriers [43, 44]. The aim of this paper is to describe a multi-level stakeholder-driven approach to co-designing a peer role for integration into community-oriented primary care teams in Cape Town, South Africa.

## Methods

### Setting

This study was conducted in low-income communities within the Eastern, Khayelitsha, and Klipfontein subdistricts within Cape Town. These subdistricts are characterized by high rates of poverty, HIV, crime, and SU. Free HIV testing and treatment is available at community health clinics, which oversee community-oriented primary care teams and other HIV services provided by CHWs. The Western Cape Department of Health contracts NGOs to employ CHWs and operate community-oriented primary care teams [5]. Although SU treatment options are limited, there are free or low-cost SU outpatient programs available in these districts [45].

### Design

**Fig. 1:**
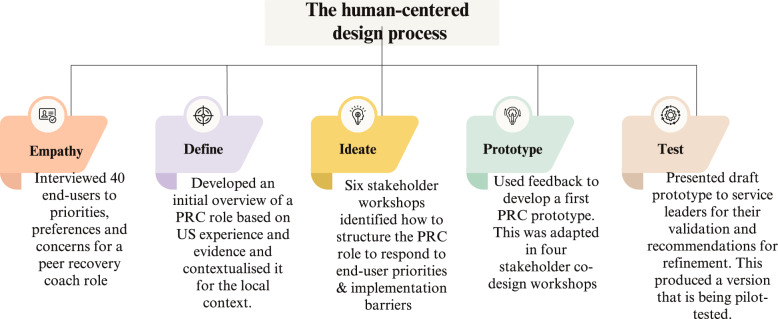
The process of designing a peer recovery coach role for community-oriented primary care teams in South Africa

We chose human-centered design as our design method as it facilitates the rapid implementation of health system and workforce innovations [42, 46]. This approach seeks to understand the implementation context and stakeholders’ design priorities and concerns [47], actively involving stakeholders in the design process [48]. Our approach involved five steps [49] shown in Fig. 1: (i) *empathizing* with stakeholders by understanding their priorities and concerns about the proposed role; (ii) *defining* the peer role and identifying information gaps; (iii) *ideation* of the peer role and function; (iv) co-designing a peer role *prototype*; and (v) *testing* this prototype via stakeholder consultation, in preparation for pilot implementation of the prototype. As this was an iterative process, this paper describes the methods involved for each step in the order in which they occurred. Although a detailed description of Step 1 has been previously published [22], a summary of methods and findings are presented here to allow for a comprehensive description of the design process. However, the focus of this paper is on describing Steps 2 through 5, which build upon and extend the findings from Step 1.

### Participants and procedures

#### Step 1: exploring stakeholders’ priorities for a peer role

Procedures for *Step 1* are described in Magidson, Rose et al. [22]. From February to June 2021, 40 semi-structured interviews were conducted (*n* = 15 PWH; *n* = 25 healthcare workers; Table 1). The Western Cape Department of Health assisted in identifying healthcare workers who were at least 18 years old and either provided or managed community-based HIV services, provided clinic-based services but interacted with community-based teams, or provided SU treatment. Healthcare workers identified PWH who were ≥ 18 years old and self-reported difficulties with HIV care engagement and SU.

**Table 1:** Characteristics of stakeholders participating in the co-design process

	Interviews (Step 1)		Workshops (Steps 3 & 4)	
	*Health care worker* *n* = 25 n (%)	*Patients* *n* = 15 n (%)	*Health care worker* *n* = 12 n (%)	*Patients* *n* = 12 n (%)
Age—M (SD)	41.2 (8.8)	40.1 (8.2)	42.8 (15.4)	37.5 (8.7)
Female	17 (68%)	11 (73%)	9 (75%)	7 (58%)
Race				
Black African	10 (40%)	15 (100%)	6 (50%)	8 (67%)
Coloured^a^	12 (48%)	0	5 (42%)	4 (33%)
Other^b^	3 (12%)	0	1 (8%)	0
Highest Education—n (%)				
< Secondary	3 (12%)	8 (53%)	0	9 (75%)
Secondary	7 (28%)	5 (33%)	2 (17%)	3 (25%)
Some Tertiary	8 (32%)	1 (13%)	4 (34%)	0
University Degree	7 (28%)	1 (13%)	6 (50%)	0
Years in Current Role *(Health care worker)*—n (%)				
< 1 year	1 (4%)	–	4 (33%)	–
1 to 3 years	9 (36%)	–	4 (33%)	–
> 3 years	15 (60%)	–	4 (33%)	–
Employment Status *(Patients)*—n (%)				
Unemployed	–	10 (67%)	–	9 (75%)

^a^Coloured is an official South African racial category referring to people of mixed-race ancestry who have a unique cultural identity

^b^Other includes participants who identified as White, Indian, or Asian

Trained research assistants obtained written informed consent before using a semi-structured interview guide to enquire about SU support needs, acceptability of using peers to address these needs, and potential roles for peers in HIV care teams. Interviews were conducted in isiXhosa or English, lasted approximately 45 min, and were audio-recorded, translated into English (if required), and transcribed verbatim.

Guided by thematic analysis [50], we used a hybrid deductive-inductive approach to code the transcripts and develop themes [51]. The interview guide was used to develop an initial codebook deductively, with inductive codes added through the open coding of several transcripts. The remaining transcripts were coded by two independent coders, who met weekly to review codes and resolve discrepancies.

#### Step 2: outlining the peer role

In February 2022, a US-based design team was formed, comprising (i) a certified peer recovery coach supervisor with experience working as a peer, and (ii) two US-based research assistants with experience working on US-based studies employing peers and studies based in South Africa. First, this team met with the study’s US-based project manager to learn more about the study context and brainstorm how typical functions of the PRC role in the US could inform a South African-based peer role.

Next, drawing from their experience, best available evidence, and US-based peer training materials, this team developed an overview of US-based peer role requirements and functions. In the State of Maryland, where the US team is based, peer recovery coaches are defined as trained and often state-certified individuals with lived experience with substance use and recovery. In this state, individuals in SU recovery for two or more years are eligible to apply for peer certification which requires completion of specialized training in Advocacy, Ethical Responsibility, Mentoring/Education, and Recovery/Wellness, and supervised practice hours. Typical functions of this role in the US include provision of psychosocial support, motivation for recovery, sharing of relevant lived experiences, and service navigation.

In March 2022, this overview was presented to the study’s operations team, comprising three SA-based research assistants (with experience working as a peer or non-clinician interventionist on South African-based studies), the South African- and US-based project managers, and a US-based clinical psychology doctoral trainee. Their feedback was used to adapt the overview. Between March and April 2022, the design and operations teams met regularly to tailor the overview to the local context and address stakeholder concerns and priorities identified during *Step 1*. The design team also recorded a brief video where the US peer supervisor explained the peer role. This video was shown during *Step 3* workshops.

Meanwhile, the combined US-South African operations team co-developed objectives and human-centered design questions for these workshops through iterative rounds of feedback. These questions took the form of “how might we” questions and broadly focused on addressing gaps in the team’s understanding of how to define the peer role within the context of community-oriented primary care teams, how to structure peer role activities, and criteria to consider when recruiting peers.

#### Step 3: ideation workshops with stakeholders

Between April and June 2022, we conducted six ideation workshops with *n* = 12 patients (across three workshops) and *n* = 12 healthcare workers (across three workshops); see Table 1 for demographics. Recruitment and eligibility procedures followed those of *Step 1*.

South African research assistants obtained informed consent and collected participants’ demographic information before the workshops. The workshops began with an overview of the peer role and the peer video developed in *Step 2*. Next, research assistants used a semi-structured workshop guide that included the design questions to elicit feedback on the proposed role. Workshops were conducted in community centers or via online platforms in English, Afrikaans, or isiXhosa (the local languages) and lasted up to an hour. All workshops were audio-recorded, with recordings supplemented by notes taken by research assistants.

We used a rapid form of qualitative data analysis recommended when engaging in iterative co-design [52]. After the workshops, staff reviewed their notes and augmented these with workshop observations. Notes were checked against recordings and supplemented with quotes from participants. The operations team met to rapidly code these augmented notes for key themes and recommendations. To aid interpretation, feedback from all the workshops was distilled into a matrix organized by theme and type of stakeholder.

In May 2022, the project operations team met with a US-licensed clinical psychologist with experience supervising US-based peers. During a whole-day in-person meeting, this team collaborated to use this matrix to adapt the peer role overview and develop a prototype for the peer role in preparation for *Step 4*.

#### Step 4: prototype co-design workshops

In June 2022, we invited participants from Step 3 to participate in prototype co-design workshops (*Step 4*). We conducted two workshops with healthcare workers (*n* = 7) and a further two workshops with patients (*n* = 9). The workshops followed *Step 3* procedures. After presenting the peer role prototype, research assistants used a semi-structured guide to elicit general feedback on the prototype, adaptations required to enhance acceptability and feasibility, and strategies for addressing contextual barriers. Workshops were conducted in community centers or via online platforms in English, Afrikaans, or isiXhosa, lasting between 45 and 60 min. All workshops were audio-recorded, with recordings supplemented by notes. The operations team met to review this feedback, using the approach outlined in *Step 3* to rapidly adapt the peer role prototype (producing version 2) in preparation for *Step 5*.

#### Step 5: stakeholder consultation to test the prototype

Next, the operations team met with stakeholders responsible for commissioning and implementing community-oriented primary care teams and clinic-based HIV and SU services. In these consultation meetings, we presented prototype version 2 and requested these leaders to provide feedback on the prototype including suggestions for strategies to support embedding the role within community-oriented primary care teams. Research assistants took notes to summarize feedback. The team met to review these notes, using the approach outlined in *Step 3* to further adapt the peer role prototype, producing version 3.

## Results

### Acceptability of the peer role and potential functions for peers within community-oriented primary care teams (step 1)

As mentioned in the Methods, detailed findings from Step 1 have been published [22] and are only summarized here. The direct quotes from participants to illustrate these findings are new and have not been previously published. Stakeholders described high levels of SU stigma among healthcare workers, and stigma’s impact on HIV care engagement for people with SU. All stakeholders thought peers could offer a unique contribution to community-oriented primary care teams by supporting patients with SU recovery and health care navigation. Healthcare workers commented that peers could be “*role models*” for patients, showing them that “*SU recovery is possible*.” Patients said they would be more comfortable talking to peers about SU compared to healthcare workers from whom they anticipated stigma. As one patient commented:

*“I prefer someone who has had the same, has gone through what I’ve also gone through…someone that I can talk to about anything and everything.”*

Stakeholders also thought that peers could help overcome stigma and other HIV care and SU treatment engagement barriers for patients with SU. They reflected that through social contact with healthcare workers and sharing their lived experience, peers could shift healthcare workers attitudes toward patients with SU while demonstrating effective ways of engaging with them. Further, they suggested that having peers on the Community-Oriented Primary Care team may make HIV services more “*welcoming*” for people with SU.

Patients and healthcare workers had different priorities for the peer role. Patients wanted the defining feature of the role to be the sharing of lived experiences of and support for SU treatment engagement and recovery. They also prioritized tailoring the format and content of peer services to meet individual needs, prioritizing confidentiality of peer-patient interactions. Healthcare worker priorities focused on peer role clarification to avoid potential role overlap with the CHW role. They also prioritized streamlining peer activities to fit the current workflow of the community-oriented primary care team. Peer wellbeing was an additional consideration for peer role design, with healthcare workers raising the importance of peer training and supervision including strategies to manage contextual and work-related risks to their recovery. This feedback informed planning for the peer role in *Step 2*.

### Insights from ideation workshops (step 3)

Five themes were generated from the Ideation workshops: (i) peer role expectations and functions; (ii) experience and knowledge prerequisites for peer role acceptance; (iii) structure and content, (iv) location and implementation of peer-delivered sessions; and (v) essential components of peer training.

#### Peer role expectations

All stakeholders were supportive of integrating peers into community-oriented primary care teams. They thought peers would ease the workload of CHWs who were “*already doing too much*” and address an important SU service gap. These stakeholders recommended that the peer role involve patient education about SU, its effects on HIV, and SU service options; equipping patients with SU behavior change skills and supporting PWH to navigate barriers to SU and HIV services.

#### Prerequisites for the peer role

Stakeholders described relevant lived experience, personal recovery, and community knowledge as prerequisites for any peer. There was broad consensus that lived experience of SU and SU recovery was a role prerequisite. While lived experience of HIV was less salient, stakeholders emphasized the importance of ensuring that peer training included components focused on HIV. One healthcare worker shared:

*“I don’t think you only need to focus on a person that is HIV positive… I think it is a person who experienced or dealt with substance use, that is a good person to be part of the team. Because then you can speak from your own experience… that person [the peer] who dealt with it, who has the experience, can be taught [about HIV].”*

While stakeholders agreed that peers did not need to have experience with a particular type of substance, they commented on the importance of peers being trained to understand the effects of the different substances being used in their communities. As one patient noted “*Alcohol or drugs [is fine]. They must at least have training [on both]*.” Healthcare workers and patients recommended that the peer have a minimum of one year in SU recovery. They thought this general guideline would ensure that peers had sufficient personal experience of recovery to support others. Several HCWs thought peers earlier in their recovery journey would be more vulnerable for relapse when encountering open substance use scenes as they would have less established strategies for managing these environmental risks. As one healthcare worker commented,

*“I think for a year… there’s no timeline that keeps you safe… So you could be in recovery for ten years, then something, and then you’re back. I just wanted to say a year to give them some time to work on themselves.”*

While there were some dissenting voices, most stakeholders believed that peers should not work in their own neighborhood. Healthcare workers were concerned about the welfare of the peer, describing the potential for the peer to be stigmatized by their own community if their SU history became known, and for patients to visit their homes for after-hours assistance. Patients’ concerns focused on confidentiality and dual relationships, with many being more comfortable working with a peer outside of their community. However, all stakeholders agreed that the peer would need to be very familiar with the community and share its culture. They described this as a prerequisite for peer safety (and therefore role feasibility) and for acceptance by the community.

#### Peer session structure

Healthcare workers and patients recommended that the peer-delivered patient sessions to include an initial “*intensive support*” phase, characterized by weekly contact sessions lasting up to 30 min followed by a less intensive “*step-down*” phase with tapering to bimonthly and then monthly sessions. As a healthcare worker commented:

*“I would say, in the acute phase, you should obviously have more frequent visits, until you see [the patient] is working with you, cooperating, then you will have less visits…stagger it, so that at least you have a system of, “now you’re in the acute phase, but now you’re going to a less acute phase where you now see less of me and can perform and function on your own.”*

Stakeholders’ recommendations for the duration of peer contact ranged from one to six months, recommending that “*the timeframe should be tailored [to the person]*.” After discussion, they agreed it would be feasible to provide peer sessions tapering in frequency for up to three months.

Stakeholders also discussed session delivery, prioritizing face-to-face contact. While some patients recommended supplementing this with digital or telehealth contact, healthcare workers were concerned that this would over-burden the peer as they would be *“on call like 24/7.”* Stakeholders also raised concerns about confidentiality when using digital messaging services, commenting that others might view these messages as mobile phones were often shared within households.

#### Session location

Stakeholders provided suggestions for the location of peer-delivered sessions including patients’ homes, community spaces such as libraries, and clinics. Some patients worried that the peer may be uncomfortable conducting sessions in their homes, suggesting that “*the first few sessions should be at the clinic so that I can tell them about my home situation, and they can decide if they want to still come to my house*.” Others raised stigma concerns, stating that neighbors would become aware of their SU if peers conducted home visits that were in addition to routine CHW visits. As a result, stakeholders agreed that the peer should offer patients a choice of session location and that the physical and psychological safety of both the peer and patient should be considered when selecting the session location.

#### Peer training

Stakeholders highlighted knowledge and skills-based competencies to target in peer training. Stakeholders agreed that training should include topics on HIV and SU, including the effects of various substances, the impact of SU on HIV, SU treatment options and how to support patients to navigate barriers to SU behavior change and SU treatment. In addition, stakeholders training on therapeutic competencies related to confidentiality and professional ethics, non-judgmental communication, and therapeutic relationships. Patients emphasized the therapeutic relationship, saying that peers “*must bring you on a level that you feel comfortable*” and “*must be trained to build relations*.” Healthcare workers emphasized training on professional work practice, especially for peers new to the healthcare workforce. As one healthcare worker commented:

*“…the ethics and the professionalism that needs to be brought into training so that people know yes, you’re using substances, but it is not for me to go and tell the whole world that you are in the program*.”

This feedback shaped the initial peer role prototype, described in Table 2.

**Table 2:** A description of the changes made to the peer recovery coach prototype at each stage of the co-design process

Role description	Peer prototype (Version 1); produced after ideation workshops *(Step 3)*	Revised prototype (Version 2); produced after co-design workshops *(Step 4)*	Revised prototype (Version 3); produced after consultation with health leaders *(Step 5)*
Role Prerequisites	• Lived experience of SU • At least one year of SU recovery • Lived experience of HIV is not essential • Understanding of community dynamics • Share language and culture of community	*No changes*	*No changes*
Role expectations and core activities	• Provide education on HIV and SU • Support patients to connect to and navigate SU and health services • Support to enhance motivation and overcome barriers to SU and health service use	*Added*: • Sharing of personal experience to reduce stigma and instill hope for recovery	*Added:* • Participation in supervision and peer mentorship • Participation in self-care activities • Participation in community-oriented primary care team activities and meetings
Working conditions	*Not addressed*	*Added*: • Safety: peer to work in pairs or be accompanied by a CHW • Working hours and conditions identical to those of community-oriented primary care team • No after-hours contact; respond to messages on next working day • No uniform due to stigma concerns	*No changes*
Core elements of Peer training	• Confidentiality and professional ethics • Content knowledge: - SU, HIV, and how they relate - Local SU services and how these can be accessed • Basic counselling skills to support behavior change: - Motivational Interviewing - Nonjudgmental communication - Problem Solving - Behavioral Activation	*Added:* • Training on safe sharing of personal experiences of SU and SU recovery	*Added:* • Information and training on community-oriented primary care team Self-care skills to support peer well-being and recovery • Benefits of supervision and peer mentorship/debriefing
Structure and content of peer sessions	• *Structure*: - One-on-one sessions for 12 weeks - Session 1: Describe peerC role, establish confidentiality and preferences for session format and location - Weeks 1–4: 1 session per week - Weeks 5–8: 1 session every 2 weeks - Weeks 9–12: Patient and peer to decide on frequency • *Duration*: ~ 30 min, with up to an hour scheduled • *Location*: Initial contact (session one) at patient’s home, community-oriented primary care team will introduce peer, location of other visits to be decided • *Mode*: Face-to-face delivery with telephonic delivery if required	*Added:* • *Structure:* - Session 1: Establish preferences for session times. Clarify boundaries of peer role and after-hours availability. Share personal experience to engage patient - Weeks 1–4: Help identify and navigate challenges to engaging in SU/HIV care via education, sharing lived experience and teaching skills for behavior change - Weeks 5–8: Provide support for recovery and enhance motivation for care engagement, using skills described above - Weeks 9–12: Provide support for care engagement (if required). Help patient transition from the peer to other recovery supports in the community (e.g. support groups) • *Location*: Not all patients endorsed an initial home visit due to stigma and safety concerns. To enhance acceptability, the initial peer session will not occur in a separate home visit. It will be delivered during CHWs’ routine visits to the patient’s household	*No changes*
Strategies to facilitate peer integration into community-oriented primary care team	*Not addressed*	*Not addressed*	*Added:* • community-oriented primary care team training prior to integration: - Information on SU, HIV and stigma - Information on peerC role and how it can support the community-oriented primary care team - peer video - Strategies for supporting community-oriented primary care team wellness - Opportunities to discuss and resolve any concerns • Formal introduction of peer to community-oriented primary care team at a team meeting • Peer to accompany CHWs on home visits for 8–10 weeks to ensure familiarity with team activities and processes, patients, and the community and to build team relationships • Peer supervision and mentoring

### Feedback from prototype co-design workshops (step 4)

Stakeholders largely endorsed the proposed prerequisites for the peer role, role expectations, training components, and session structure. Stakeholders recommended augmenting this prototype to include strategies for managing contextual challenges like safety, a description of the peer’s working conditions, and more detail about the content of peer-delivered sessions. Table 2 presents changes to the prototype based on this feedback.

### Feedback from consultation meetings (step 5)

Stakeholders responsible for commissioning and implementing HIV and SU services endorsed the revised prototype but noted that it did not address the context of the community-oriented primary care team. HIV experts recommended expanding peer role expectations to include participation in the broader community-oriented primary care team and augmenting the proposed training components to include information on these teams. They also recommended developing peer integration training for the community-oriented primary care team to orient them to the peer role. Further, SU experts recommended augmenting both the peer role expectations and training schedule to include supervision, peer mentoring and self-care components. They described these components as necessary for helping peers navigate potential barriers to their integration within community-oriented primary care teams, complex community dynamics, and role boundaries while supporting their professional development and safety. Table 2 outlines modifications based on this feedback.

## Discussion

This paper contributes to a small but expanding literature on the value of human-centered design approaches when designing interventions for dissemination and sustainment [42, 46, 53] by demonstrating the benefits of a stakeholder-engaged approach to the design of workforce innovations that address the SU service gap in resource-constrained settings. Overall, the multiple stakeholder groups we engaged in the design process thought it was acceptable and feasible to embed peers into community-oriented primary care teams, with the caveat that the peer role should remain distinct from existing roles within the community-oriented primary care team. These stakeholders thought the peer role should focus on providing SU recovery and both SU and HIV care navigation supports for PWH—services that CHWs do not have the time, training, or capabilities to provide as reported by previous studies [16]. Despite this consensus, patients and healthcare worker stakeholders differed in their reasons for wanting the peer role to remain distinct from that of CHWs.

More specifically, patient stakeholders prioritized the relational components of the peer role, desiring a better care experience than the one they currently received from CHWs. Like earlier studies [31, 33], patients described their HIV care experience as characterized by SU stigma, poor care quality, and unsupportive provider relationships. These stakeholders hoped that peers could offer an alternative care experience by sharing their lived experience of SU recovery to build a strong patient-peer relationship, and by providing person-centered and confidential support for SU behavior change and recovery and both SU and HIV care navigation tailored to their personal treatment goals. They also wanted flexibility and choice in how sessions were structured and delivered, similar to patient and peer feedback on the essential elements of peer recovery services from US-based research [40, 54]. During the iterative design process, we used this feedback to modify the peer role prototype to better align with patients’ priorities and preferences. Modifications included (i) expanding peer role expectations and session content to foreground the sharing of lived SU experiences; (ii) integrating the initial peer visit with routine CHW visits to the patient’s home to help with initial relationship building and to address SU stigma concerns; and (iii) introducing opportunities for shared decision making about the structure and delivery of peer sessions.

In contrast, healthcare workers and other stakeholders prioritized greater definition and clarification of the peer role, including role boundaries, and suggested modifications to better align the peers work practice with that of the community-oriented primary care team. They were concerned about potential role duplication and that differences in working conditions, expectations, and practice may increase the amount and complexity of their work. These concerns are not surprising given the unpredictability and insecurity of CHW employment contracts [55] and the well-documented high workload of community-oriented primary care teams [7]. CHWs are unlikely to welcome a peer into their teams if they think this will make their role redundant or increase the complexity of their work. Role ambiguity has been identified as a barrier to embedding peers within US-based primary care [56] and non-specialist providers within primary healthcare more generally [57, 58]. In response to these concerns, we modified the peer prototype to minimize any role duplication by clarifying that CHWs would be responsible for HIV treatment support and peers would be responsible for SU-related support, including direct supports for SU behaviour change and SU recovery and supports with referrals to SU treatment and SU treatment navigation; services not currently provided by CHWs. Further modifications were made to align the peers proposed working conditions and expectations for professional work practice with those of the community-oriented primary care team, with peer training and supervision augmented to address these components.

Role clarification was not a salient concern for HIV and SU service leaders, possibly because they were consulted later in the design process. Based on their health systems experience, these stakeholders offered unique insights into the readiness of community-oriented primary care teams for peer integration. They emphasized the need for community-oriented primary care team members to be educated about the potential contributions of the peer role to address the unmet SU-related treatment needs of patients, and the importance of preparing these teams for peer integration. These stakeholders raised concerns about the potential impact on peer wellness if the climate of the community-oriented primary care team was not welcoming, suggesting that the peer may require additional mentoring and support to navigate these implementation barriers and ensure their psychological safety. These recommendations are aligned with the experiences of introducing peer services for SU and mental health in other contexts [59, 60]. Based on this feedback, we added the following components to the peer role prototype: (i) community-oriented primary care team integration training focused on raising awareness of and fostering openness to the peer role; (ii) community-oriented primary care team preparation activities to create opportunities for the peer to build relationships with team members through meetings and work shadowing; (iii) additional training components to orient the peer to the community-oriented primary care team environment and how to work effectively with this team; and (iv) clinical supervision and peer mentoring to support peers to deliver high-quality support, navigate team and community dynamics, and support their psychological safety and wellbeing in complex environments. Earlier studies also identified training and relational work with existing staff as key strategies for supporting the implementation of primary healthcare workforce innovations for mental health [61] and supervision and peer mentoring as important strategies for preventing burnout and protecting the wellbeing of non-specialist health providers in South Africa [29, 62].

While findings highlight the value of including patient, provider, and systems perspectives, engaging multiple stakeholder groups in co-design is complex and fraught with power imbalances. This study included patients who were marginalised by their socio-economic status, HIV and SU, and CHWs with little power or agency in the healthcare system [5]. Further, there were power imbalances within our study team, inherent when adapting an intervention from the global North for application in the global South, and where teams include people with lived experience of SU and other stigmatized identities. To limit the impact of these imbalances on stakeholder inputs, we intentionally used mutual capacity-building (which promotes equitable bidirectional learning) as a guiding framework for stakeholder engagement [63, 64]. In all team and stakeholder activities, we worked to ensure that all contributions were equally valued, that everyone felt respected, and encouraged a diversity and divergence of perspectives. We distributed leadership and decision-making power across teams and groups, encouraging open discussion, and preferencing stakeholder contributions and the local voice in design decisions over those of the study team. Given the iterative design process, we could demonstrate how we used stakeholders’ feedback to modify the prototype. Building stakeholders’ trust and investment in the design process and enhancing the quality of their contributions. With this approach enriching our design process, we recommend mutual capacity building as a framework for supporting stakeholder engagement in the co-design of SU interventions.

Despite this strength, there are limitations to consider. A relatively small number of patients and healthcare workers participated in the workshops. Additional engagement with a wider range of patients and healthcare workers may identify further modifications to the peer role that could broaden its appeal and utility. Second, the peer role was designed to be embedded in community-oriented primary care teams working within disadvantaged communities in the Cape Town metropole where there are high levels of unmet SU-related needs and structural barriers to accessing SU treatment [65]. Inter-provincial differences in the organization, function, and funding of community-oriented primary care teams and SU treatment needs [5, 9] may affect the relevance and utility of the peer role beyond the current context. Third, we did not quantitatively assess acceptability, feasibility, or appropriateness at each step of the co-design process. Future studies should consider embedding quantitative implementation outcome measures into the co-design process so that the potential utility of the co-design process for enhancing acceptability, feasibility, and appropriateness and future implementation of this role can be systematically evaluated.

## Conclusion

This paper describes a methodological process that may be useful to other teams developing lived experience and peer roles for primary healthcare teams. Through engaging stakeholders with diverse perspectives in the design process, we were better able to understand and respond to their priorities and preferences for the peer role, enhancing role acceptability and contextual fit. Stakeholder engagement identified patient-, provider-, and systems-level barriers to role implementation; the iterative design process allowed us to modify the PRC prototype to enhance role feasibility and contextual fit. Stakeholder engagement also served as an implementation strategy, building collective ownership in the peer role and helping us identify champions to support the pilot implementation of this workforce innovation. A pilot study (NCT05907174) is underway to evaluate the acceptability and feasibility of embedding the peer role within community-oriented primary care teams and preliminary effects on HIV care and SU treatment engagement. While findings from this pilot are likely to inform further modifications to the peer role prototype, we believe investment in this stakeholder-driven approach to the design of this peer role will enhance the likelihood of future scaling and sustainment of this role within the SU workforce and can offer a broad methodological approach to enhancing the acceptability and feasibility of other SU workforce innovations.

## Data Availability

The datasets generated and analysed during the co-design process are not publicly available due to regulatory restrictions on the public availability of research participants’ data. De-identified data are available from the corresponding author on reasonable request.
